# Foreign Bodies in the Air Passages

**Published:** 1859-05

**Authors:** Charles Brackett

**Affiliations:** Rochester, Ind.


ARTICLE III.

### FOREIGN BODIES IN THE AIR PASSAGES.
### CASE OF INTESTINAL CONCRETION.

BY CHARLES BRACKETT, M. D., ROCHESTER, IND.

The following cases of foreign bodies in the air passages, which were never removed by surgical interference, are somewhat interesting:

July 8th, 1852.—John Hochheim, of Marshall county, brought to me his boy, some two or three years of age, who not long before had got a German copper coin, of about the value of one-third of a cent—a kreutzer, I think, it is called—into the trachea. The patient was troubled with occasional attacks of suffocation, spasmodic cough, and suffering considerably for breath. I could detect by auscultation the seat, or location of the coin in the right bronchus. The mother would not consent to have tracheotomy performed. I directed quiet, and general attention to health telling what results might follow. Some weeks later, they brought the child again, willing that an operation might be performed. The coin had ulcerated its way down in the lung, so that I could, or did not recommend an operation. I saw the child occasionally after that, for a couple of years, when his health became better, and finally quite good, as I am informed. The patient is now living in Plymouth, Marshall county, in tolerable health.

*Case II.*—L. E. Rannells, of Rochester, some five years since, during my absence from town, got a grain of burned coffee in the lung, through the glottis. It found its way low down, filled one of the bronchi, produced severe constitutional disturbance, finally became fixed, and, I believe, is yet in the lung. The boy is healthy, but has grown in weight, or stature, scarcely at all, since the accident. I examined him frequently, and for a long time, could tell very nearly the precise location of the kernel, but, for the past two or three years, have not auscultated him.

*Case III.*—Shortly after harvest, last fall (1858), a man came to me, from the edge of Marshall county. His name I

did not take. Said his boy, some nine years of age, had got a water-melon seed in the wind-pipe. I told him what I could do for him, or what nature *might* do. He went home, consulted his wife, and concluded to let nature do her best. I saw the man week before last, who said that, during a severe attack of coughing, the melon seed, much decayed, was thrown from the wind-pipe of the boy with considerable of pus, and the patient was then quite hearty; that, for a long time, he tried to induce his wife to have an operation performed, but she preferred leaving the case to nature, and both believed he would die.

These three cases, I believe, could all have been speedily relieved by an operation shortly after the reception into the trachea of the foreign bodies. Yet they all, ultimately, did well, so far as they have progressed. I shall endeavor to keep watch of the two cases where the offending cause is not yet removed.

I saw the following case of intestinal concretion, April, 1849: Mrs. Wertz, in labor, as she and her attending physician supposed, for the past twenty-four hours before I saw her. A vaginal examination revealed to me that the rectum was impacted with a mass as nearly as large as a child's head. I gave an injection of inspissated ox gall, dissolved in warm water; after a short time, the woman became easy, comparatively speaking, got up to stool, and cried out that it was coming (meaning the child). I told her to bear down, which she did, and was delivered of a mass, about five inches in diameter. It was composed, I thought, mostly of magnesia, with fecal matter. She had been in the habit of eating carb. magnesia freely, for the relief of pyrosis and gastrodynia, was an inveterate opium eater; had had for some time previons a sort of dysenteric diarrhœa, and could not believe that there was any obstruction in the bowels. Two weeks afterwards, she was safely delivered of a healthy child, and became hearty, as usual for her. At that time, I was rather too fastidious, or over nice, to analyze, or measure carefully, a fecal mass of that kind, and it was thrown out. It was in size, however, fully as large as the head of a fetus at full time, as nearly as I could judge from ocular measurement.

[REMARKS.—We have taken the liberty of publishing the case of intestinal concretion, which, probably, was not intended for that purpose by Dr. Brackett.

In reference to the cases of foreign bodies in the air passages, while they show the power of the lungs to resist the deleterious action of substances so placed in certain cases, and although many similar cases may be found on record, they do not by any means justify the practice of deferring an operation, if the surgeon be called upon soon after the accident. The operation of tracheotomy for a foreign body, is not, in general, a dangerous one, if performed immediately. If deferred until inflammation has been developed, the result is often fatal.

How far a surgeon might be justified in advising against an operation when the foreign body is metallic, and the air passages have become so habituated to its presence that little inconvenience is felt, it is impossible to determine, such cases being rare. Metallic produce less irritation than non-metallic bodies, and when they become so imbedded as to be no longer moveable, it is obvious than an operation would not be advisable. The practice of Dr. Brackett seems to have been in accordance with this view, and was successful.—ED.]

